# Preference of *Proteomonas sulcata* anion channelrhodopsin for NO_3_^−^ revealed using a pH electrode method

**DOI:** 10.1038/s41598-021-86812-z

**Published:** 2021-04-12

**Authors:** Chihiro Kikuchi, Hina Kurane, Takuma Watanabe, Makoto Demura, Takashi Kikukawa, Takashi Tsukamoto

**Affiliations:** 1 Division of Soft Matter, Graduate School of Life Science, Hokkaido University, Sapporo, 060-0810 Japan; 2 Division of Macromolecular Functions, Department of Biological Science, School of Science, Hokkaido University, Sapporo, 060-0810 Japan; 3 Faculty of Advanced Life Science, Hokkaido University, Sapporo, 060-0810 Japan; 4 Global Station for Soft Matter, Global Institution for Collaborative Research and Education, Hokkaido University, Sapporo, 001-0021 Japan

**Keywords:** Biochemistry, Ion channels

## Abstract

Ion channel proteins are physiologically important molecules in living organisms. Their molecular functions have been investigated using electrophysiological methods, which enable quantitative, precise and advanced measurements and thus require complex instruments and experienced operators. For simpler and easier measurements, we measured the anion transport activity of light-gated anion channelrhodopsins (ACRs) using a pH electrode method, which has already been established for ion pump rhodopsins. Using that method, we successfully measured the anion transport activity and its dependence on the wavelength of light, i.e. its action spectra, and on the anion species, i.e. its selectivity or preference, of several ACRs expressed in yeast cells. In addition, we identified the strong anion transport activity and the preference for NO_3_^**−**^ of an ACR from a marine cryptophyte algae *Proteomonas sulcata,* named *Psu*ACR_353. Such a preference was discovered for the first time in microbial pump- or channel-type rhodopsins. Nitrate is one of the most stable forms of nitrogen and is used as a nitrogen source by most organisms including plants. Therefore, *Psu*ACR_353 may play a role in NO_3_^**−**^ transport and might take part in NO_3_^**−**^-related cellular functions in nature. Measurements of a mutant protein revealed that a Thr residue in the 3^rd^ transmembrane helix, which corresponds to Cys102 in *Gt*ACR1, contributed to the preference for NO_3_^**−**^. These findings will be helpful to understand the mechanisms of anion transport, selectivity and preference of *Psu*ACR_353.

## Introduction

In living cells, there are several kinds of proteins that transport a variety of ions and small molecules across cell membranes. The membrane transport of those substrates is directly or indirectly connected to a variety of life phenomena, such as the maintenance of cell homeostasis, cell signaling, the generation of action potential in neurons, and so on. Therefore, membrane transport proteins are physiologically important and their molecular properties, including their structures and functions, have been intensively investigated by many research groups. In this study, we focused on one type of ion transporter, ion channels.

Ion channels passively transport ions according to the electrochemical potential gradients of ions across cell membranes. No external energy is needed for that type of transport, however some factors, such as membrane potential change, binding of ligands as well as physical or mechanical stimuli, are necessary to open and close those channels. In 2002, the first light-gated ion channel-type microbial rhodopsin for cations (cation channelrhodopsin, abbreviated as CCR) was reported, which originated from a green algae *Chlamydomonas reinhardtii* and was named channelrhodopsin-1 (abbreviated as ChR1)^1^. As the name suggested, channelrhodopsins belong to a family of microbial rhodopsins, each composed of a seven transmembrane α-helical protein opsin and a chromophore all-*trans*-retinal^2^. A year later, another CCR that originated from the same algae, named channelrhodopsin-2 (abbreviated as ChR2), was reported^3^. Both ChR1 and ChR2 transport not only H^+^ but also other monovalent cations, such as Li^+^, Na^+^ and K^+^^1,3,4^. In 2015, natural light-gated anion channelrhodopsins (abbreviated as ACRs) that originated from a cryptophyte algae *Guillardia theta*, named *Gt*ACR1 and *Gt*ACR2, were reported^5^. Both of those ACRs are capable of transporting monovalent anions, such as F^**−**^, Cl^**−**^, Br^**−**^, I^**−**^ and NO_3_^**−**^^5^. Since then, many more CCRs and ACRs have been identified, characterized and engineered^6–26^. Especially for ACRs, they are novel proteins that have just been discovered and therefore their molecular characteristics and their biological roles remain to be clarified.

The molecular functions of ion channels have been investigated using several experimental techniques. Especially, electrophysiological methods, such as the patch-clamp method, are highly quantitative and accurately characterize ion channel functions. In cases of not only ACRs but also CCRs, a series of electrophysiological analyses, such as current–voltage relationship and ion selectivity or preference, have been precisely performed. In addition, the introduction of a short-pulsed laser as an actinic light source into the electrophysiological measurement system enables the measurement of transient current changes during a single sequential photochemical reaction of microbial rhodopsins, called the photocycle^2,16,17,20,27,28^. This technique is beneficial for identifying which photo-intermediates facilitate ion conducting and non-conducting states in the photocycle. Therefore, electrophysiological analysis is powerful and necessary to deeply understand the ion channel mechanisms of ACRs and CCRs. However, in exchange for such accuracy, complex instrumental setups and experienced operators are required. This is a bottleneck not only for non-experts in electrophysiology but also for scientists who want to measure ion transport activity more simply and easily.

We conceived the idea to measure the anion transport activity of ACRs using the pH electrode method, which had already been established for ion pump-type microbial rhodopsins expressed as recombinant proteins in *Escherichia coli* cells. Simple instrumental setups and usability of the pH electrode method are a great advantage although the method is less quantitative compared to the electrophysiological method. In addition, the pH electrode method is expected to easily measure the dependence of the activities of ACRs on the wavelength of excitation light, i.e. action spectra, and on the anion species, i.e. selectivity or preference, even if precise measurements for reversal potentials, current density, kinetics, and so on, are impossible to perform. However, *E. coli* cells cannot be used as host cells to express a variety of recombinant ACRs except for *Gt*ACR2^29–31^. In most cases so far, insect, mammalian and yeast cells have been used as host cells for the expression of recombinant ACRs. Among them, yeast cells are as easy to culture as *E. coli* cells and to exchange buffers for the measurement of anion transport activity. Therefore, the pH electrode method is expected to be applicable for ACRs expressed in yeast cells to measure anion transport activity and anion dependence in a relatively simple way.

In this research study, we first developed and demonstrated the feasibility of the pH electrode method to measure the anion transport activity of ACRs expressed in yeast cells. We used *Gt*ACR1, which is a well-studied ACR at present, to test the usefulness of the system. The action spectrum and anion dependence of the activity of *Gt*ACR1 measured using the pH electrode method were almost the same as previously measured using the electrophysiological method except for F^**−**^ and SO_4_^2**−**^ probably due to the difference in the experimental condition^5^. To apply this method to other ACRs, we measured the anion transport activities of 7 ACRs that were successfully expressed in yeast cells. As a result, we found that an ACR that originated from a marine cryptophyte algae *Proteomonas sulcata*, previously named as *Psu*ACR_353^19^, showed the strongest activity among the ACRs that we tested and preferably transported nitrate (NO_3_^**−**^) to other monovalent anions, such as Cl^**−**^ and Br^**−**^. NO_3_^**−**^ is one of the most stable forms of nitrogen on earth and is used as a nitrogen source by most organisms including plants^32^. Therefore, the light-gated *Psu*ACR_353 may play a role in NO_3_^**−**^ transport and might possibly take part in nitrate sensing and signaling, and furthermore, the nitrogen assimilation by *P. sulcata* in nature. Measurement of a mutant protein revealed that the Thr108 residue in the 3^rd^ transmembrane helix, which corresponds to Cys102 in *Gt*ACR1, contributed to the NO_3_^**−**^ preference in *Psu*ACR_353. These findings will be helpful to understand the mechanisms of anion transport, selectivity and preference of *Psu*ACR_353.

## Results

### Concept of the pH electrode method

**Figure 1:**
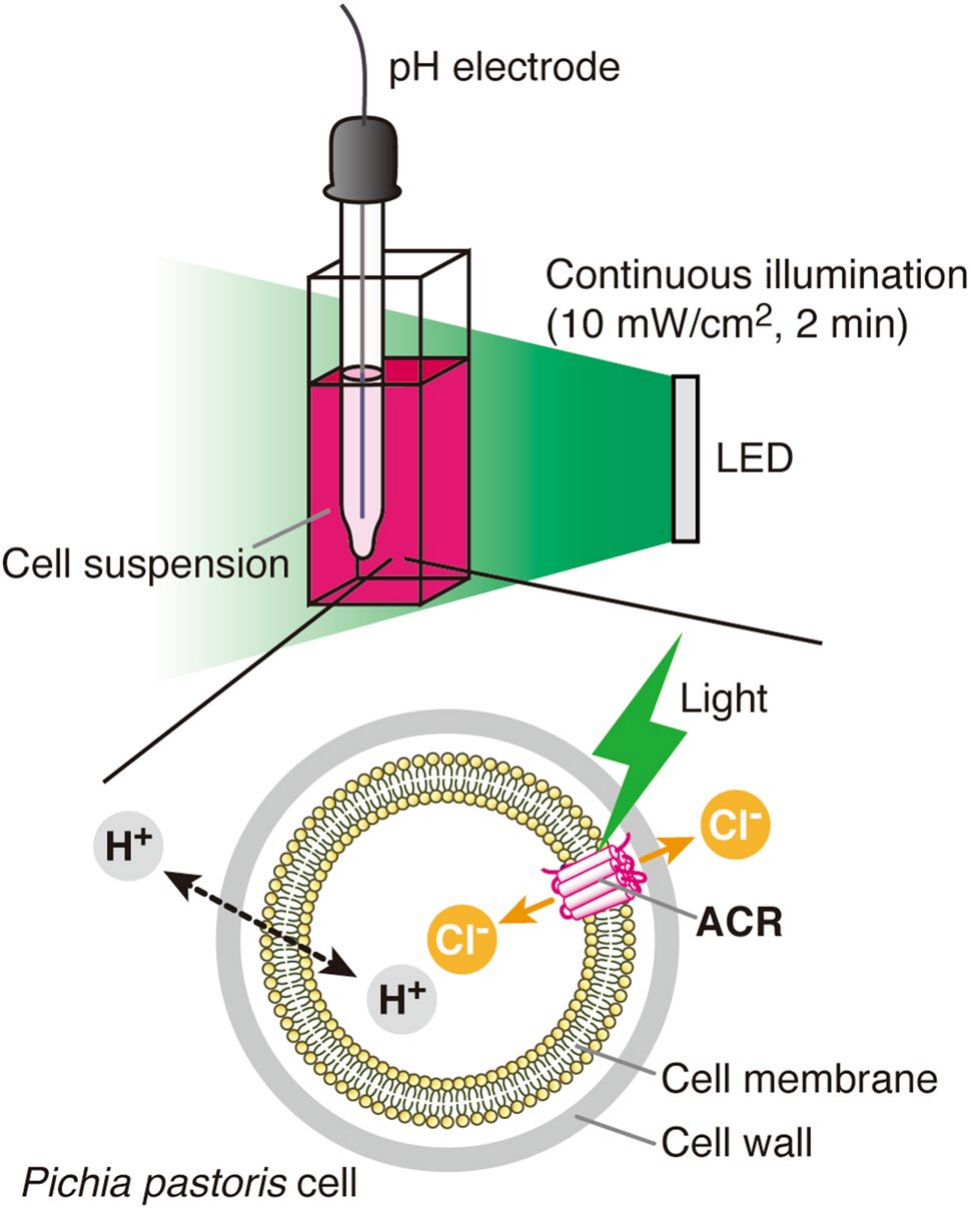
Illustration of the concept of the pH electrode method for measuring anion transport activity of ACRs expressed in yeast *P. pastoris* cells. In this system, ACRs import anions according their electrochemical potential gradient because the anion concentration outside the cells was kept higher (300 mM) than inside (nearly 0 mM). Therefore, a pH increase will be detected due to the secondary influx of protons to compensate the membrane potential being more negative due to the influx of anions.

Figure 1 illustrates the concept of the pH electrode method for measuring the anion transport activity of ACRs expressed in yeast cells. The fundamental concept was almost the same as that for the system using *E. coli* cells. The yeast cell suspension was poured into the glass vial container and the pH electrode was placed into it. The cell suspension was stirring during the measurement to keep the suspension homogeneous. Continuous LED light (10 mW/cm^2^ on average) was illuminated for 2 min from the side of the vial container. To reduce undesirable artifacts, we were careful not to let the light directly hit the light-sensitive part of the pH electrode.

Yeast cells have a cell wall outside and a cell membrane inside and ACRs are localized in the cell membrane. Once the light activated ACRs, anions were passively transported into the cells due to the electrochemical potential gradient of anions being higher outside the cells (300 mM) than inside (nearly 0 mM). This anion influx resulted in making the membrane potential more negative. To compensate for that, protons (H^+^) in the bulk solution penetrate into the cells by passing through the cell wall and cell membrane. As a result, we can indirectly observe anion transport using the pH electrode as a pH change of the suspension. In this study, we observed pH increases that reflect the anion transport into yeast cells.

### Demonstration of the pH electrode method to measure the activity of *Gt*ACR1

**Figure 2:**
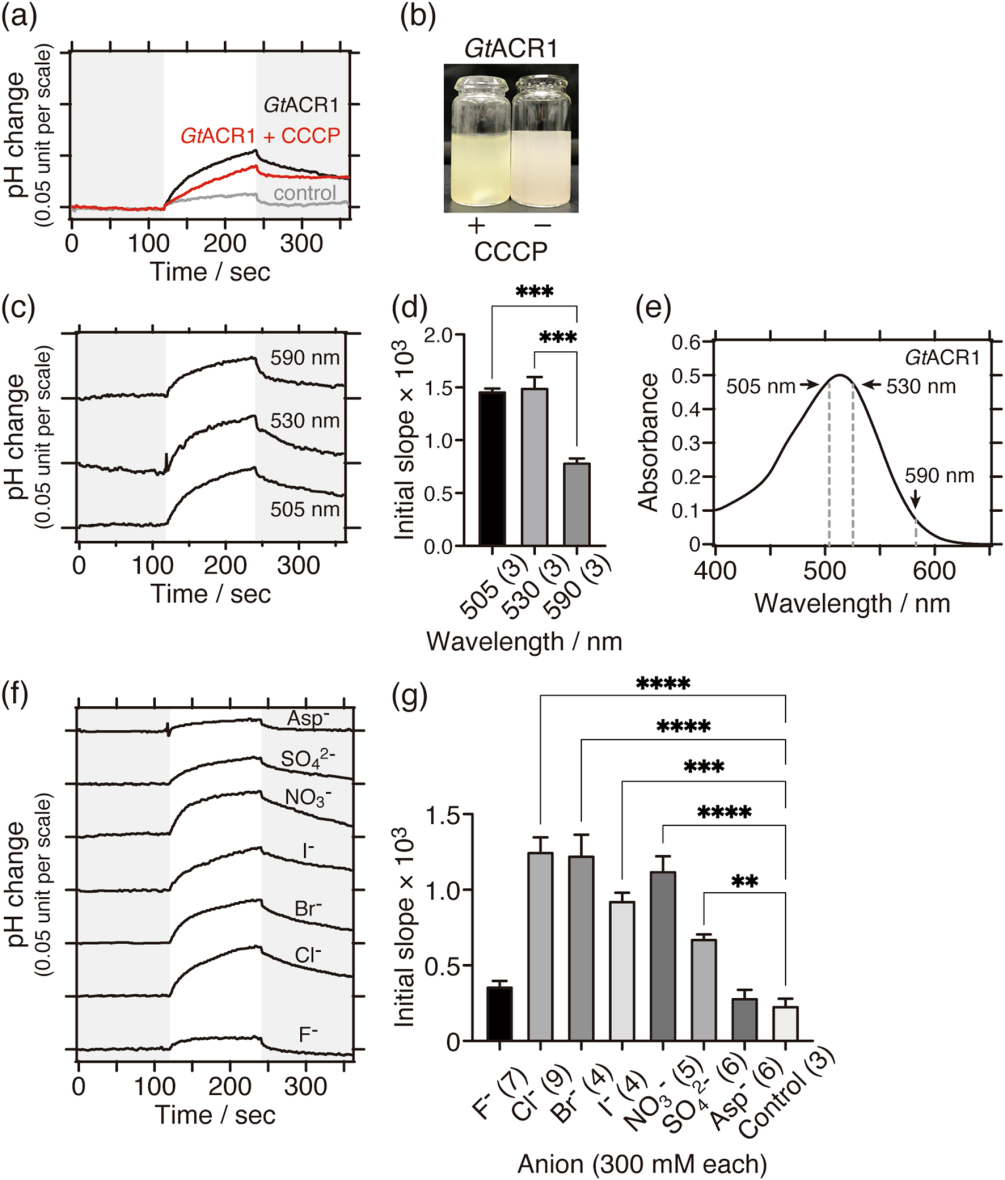
Anion transport activity of *Gt*ACR1 measured using the pH electrode method. (**a**) Comparisons of Cl^**−**^ transport activities in the absence (black line) or presence of 10 μM CCCP (red line); the negative control (grey line) was yeast cells without integration of the *Gt*ACR1 gene. 530 nm LED light (10 mW/cm^2^) was illuminated for 2 min as shown on a white background. (**b**) The color of pigmented yeast cell suspension expressing *Gt*ACR1 changed from pink to yellow by adding 10 μM CCCP. (**c**) Comparisons of Cl^**−**^ transport activities at various excitation light wavelengths (10 mW/cm^2^). LED lights were illuminated for 2 min as shown on a white background. (**d**) Statistical comparisons of Cl^**−**^ transport activities at various excitation light wavelengths. Data are reported as means and S.E.M.; the numbers in parentheses indicate the number of independent experiments. One-way ANOVA followed by Tukey’s test was performed (*p* value; *** < 0.0006). (**e**) Visible absorption spectrum of purified *Gt*ACR1 suspended in 10 mM MOPS (pH 7) buffer containing 1 M NaCl and 0.05% *n*-Dodecyl-β-D-maltoside (DDM). (**f**) Comparisons of anion-dependent transport activities. 530 nm LED light (10 mW/cm^2^) was illuminated for 2 min as shown on a white background. (**g**) Statistical comparisons of anion-dependent transport activities. Data are reported as means and S.E.M.; the numbers in parentheses indicate the number of independent experiments. One-way ANOVA followed by Dunnett’s test was performed (*p* values; **** < 0.0001, *** 0.0001, ** 0.0084). The negative control was yeast cells without integration of the *Gt*ACR1 gene resuspended in 300 mM NaCl.

To ensure that the system works, we used *Gt*ACR1 as a test sample. First, we successfully expressed *Gt*ACR1 in yeast *P. pastoris* cells according to previous studies^28,33^. We then measured the Cl^**−**^ transport activity of *Gt*ACR1 using the pH electrode method, and as shown in Fig. 2a, the activity of *Gt*ACR1 was successfully measured. The signal for the pH increase was clearly larger than that of control cells, in which no ACR gene was integrated. In most cases for anion and sodium-ion pump rhodopsins, the measurements were carried out in the presence of a proton-selective ionophore, carbonyl cyanide m-chlorophenyl hydrazone (abbreviated as CCCP), which facilitates the proton influx into cells. As a result, the signal of the pH change becomes larger than that in the absence of CCCP. Conversely, this tendency is evidence for rhodopsins being capable of transporting anions or sodium ions but not protons. However, in the case of *Gt*ACR1, the signal intensity of the pH change was decreased in the presence of CCCP (Fig. 2a) although protons are not transport substrates of *Gt*ACR1^5^. As shown in Fig. 2b, the color of the cell suspension was changed from pink to yellow, indicating that something undesirable happened in the presence of CCCP. Therefore, all subsequent measurements were carried out in the absence of CCCP. At present, however, we have no idea that explains why the signal has been decreased in the presence of CCCP. As the absorption spectra of purified *Gt*ACR1 shows (Supplementary Figure S3), the CCCP little affects the visible absorption property of active form *Gt*ACR1 (at around 515 nm), meaning that the CCCP or its solvent ethanol, whose concentrations are very low (10 μM and 0.001%(v/v), respectively), do not significantly induce the denaturation of *Gt*ACR1 and the deprotonation of the retinal protonated Schiff base.

We then measured the dependence of the excitation light wavelength on the Cl^**−**^ transport activity, i.e. the action spectrum (Fig. 2c). Stronger signals were obtained when the sample was illuminated using 505 nm and 530 nm light than by using 590 nm light. The amplitudes of the initial slope obtained from each condition were averaged and were statistically compared (Fig. 2d). As a result, the activities at 505 nm and 530 nm were not significantly different but were stronger than that at 590 nm. This result correlated well with the absorption spectrum of purified *Gt*ACR1 (Fig. 2e) and with the action spectrum previously measured using the electrophysiological method^5^. Therefore, the pH electrode method can be used to investigate the action spectra of ACRs.

The anion-dependent transport activity of *Gt*ACR1 was also measured using 530 nm LED light for activation. Six monovalent anions (F^**−**^, Cl^**−**^, Br^**−**^, I^**−**^, NO_3_^**−**^ and aspartate (abbreviated as Asp^**−**^)) and one divalent anion (SO_4_^2**−**^) at a concentration of 300 mM were tested (Fig. 2f,g). As a result, significant transport activities were obtained for the anions except for F^**−**^ and Asp^**−**^ compared to the negative control cells. As suggested by a previous electrophysiological study^5^, *Gt*ACR1 transports wide variety of anions and thus has less anion selectivity. Therefore, the pH electrode method in this study closely reproduced such an anion dependence. Therefore, we concluded that the pH electrode method can potentially be applied to measure anion transport activity, the action spectrum and the anion dependence of ACRs expressed in yeast cells.

Closely looking at the anion-dependent transport activity of *Gt*ACR1 measured using the pH electrode method, our results showed that *Gt*ACR1 transported SO_4_^2**−**^ but not F^**−**^ (Fig. 2f,g), which was opposite of the previous electrophysiological study^5^. The exact reason was unclear, however there were some differences in the experimental conditions, for example pH condition, between the two methods. In our case, the initial pH was adjusted to around 5 on average. On the other hand, in the previous electrophysiological study^5^, the pH was estimated to be from neutral to weakly alkaline due to the use of HEPES buffer. We checked the effect of pH on the Cl^**−**^ transport activity of *Gt*ACR1 (Supplementary Fig. 4). As a result, the maximum activity was obtained at pH 5, which was about 2.5-times larger than that at pH 7. It could be a reason that the dissociation state of certain amino acid residues in *Gt*ACR1 are different under the different pH conditions in each experiment. This may result in the difference in the transport activity for F^**−**^ and SO_4_^2**−**^ measured by each method.

### Application of the pH electrode method to other ACRs

The pH electrode method was then used to test other ACRs. At present, several ACR genes have been identified by transcriptome analysis. Among them, we tested 8 ACRs (3 from *Proteomonas sulcata* and 5 from *Geminigera cryophila*; see Table 1) for functional expression in yeast cells. Five of the 8 ACRs tested in this study belong to the Cys-type (see below) as does *Gt*ACR1, 2 belong to the Thr-type as does RapACR reported recently^19^, and 1 belongs to the Val-type as does *Gt*161302 reported previously^5^. Note that *G. cryophila* has 5 ACRs that belong to all three types.

**Table 1:** Gene and amino acid information of ACRs used in this study.

Protein name abbreviation	Organism	Accession	68th residue in *Gt*ACR1	102th residue in *Gt*ACR1	Number of amino acids
*Gt*ACR1	*Guillardia theta* CCMP2712	KP171708	Glu	Cys	295
*Psu*ACR1	*Proteomonas sulcata* CCMP704	KF992074	Glu	Cys	291
ZipACR		KX879679	Glu	Cys	314
*Psu*ACR_353		MG831189	Glu	Thr	300
*Gc*ACR_145	*Geminigera cryophila* CCMP2564	KX879675	Glu	Cys	326
*Gc*ACR_197		MG831184	Glu	Val	332
*Gc*ACR_201		MG831185	Glu	Thr	288
*Gc*ACR_439		KX879676	Glu	Cys	340
*Gc*ACR_457		KX879674	Glu	Cys	316

Data taken from^5,16–19^.

**Figure 3:**
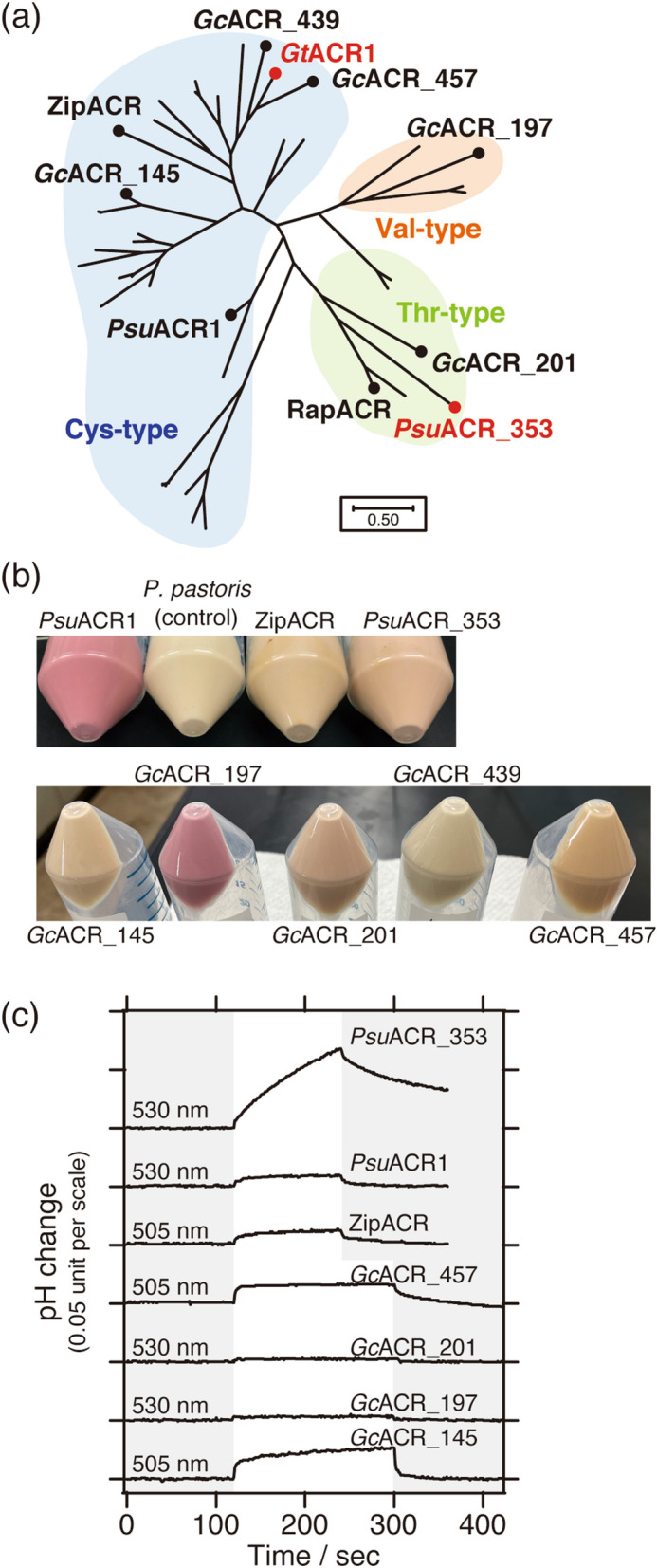
ACRs tested using the pH electrode method. (**a**) Phylogenetic relationship of 45 ACRs analyzed by MEGA 7 software. According to amino acids conserved at position 102 in *Gt*ACR1, ACRs are classified into three types, Cys-type (blue), Thr-type (green) and Val-type (orange). (**b**) Pictures of pigmented yeast cells due to the functional expression of each ACR in the presence of all-*trans*-retinal. The negative control was yeast cells without integration of any ACR gene. (**c**) Comparisons of Cl^**−**^ transport activities of ACRs. LED lights were illuminated for 2 or 3 min as shown on a white background.

ACRs can be roughly classified into three types, the Cys-type, the Thr-type and the Val-type (Fig. 3a). One of the factors contributing to that classification is the amino acid conserved at position 102 in *Gt*ACR1. It has been reported that two amino acid residues contribute to the channel gating of *Gt*ACR1^27,28^. One is Glu68 located in the 2^nd^ transmembrane helix that controls slow-opening/fast-closing gates (Table 1 and Supplementary Fig. S1). Another is Cys102 located in the 3^rd^ transmembrane helix that regulates fast-opening/slow-closing gates. The Glu residue is completely conserved in all ACRs we tested, whereas the Cys residue is substituted with Thr or Val (Table 1, Fig. 3a, and Supplementary Fig. S1). For the case of the recently reported RapACR, the Thr residue corresponding to Cys102 in *Gt*ACR1 was also shown to contribute to the channel gating kinetics^19^. However, the effect of such diversity in the amino acid conservation on anion transport activity, anion selectivity or preference is not well understood. No anion transport activity was reported for *Gt*161302, which has a Val residue corresponding to Cys102 in *Gt*ACR1^5^.

As shown in Fig. 3b, 7 of the 8 ACRs, except *Gc*ACR_439, were pigmented in the presence of all-*trans*-retinal, indicating their successful expression in yeast cells. We then measured the Cl^**−**^ transport activities for these ACRs using the pH electrode method. For activation, 505 nm LED light was used for ZipACR, *Gc*ACR_145 and *Gc*ACR_457, while 530 nm LED light was used for *Psu*ACR1, *Psu*ACR_353, *Gc*ACR_197 and *Gc*ACR_201, by taking into account the colors of the cells and the action spectral maxima reported previously^16–19^. The intensity of light was kept at an average of 10 mW/cm^2^. As a result, the signals of pH change were obtained in most ACRs but the intensities were very weak (Fig. 3c). However, only *Psu*ACR_353 generated a strong signal of a pH increase, indicating its significant Cl^**−**^ transport activity compared to the other ACRs.

### Characterization of the anion transport activity of *Psu*ACR_353

**Figure 4:**
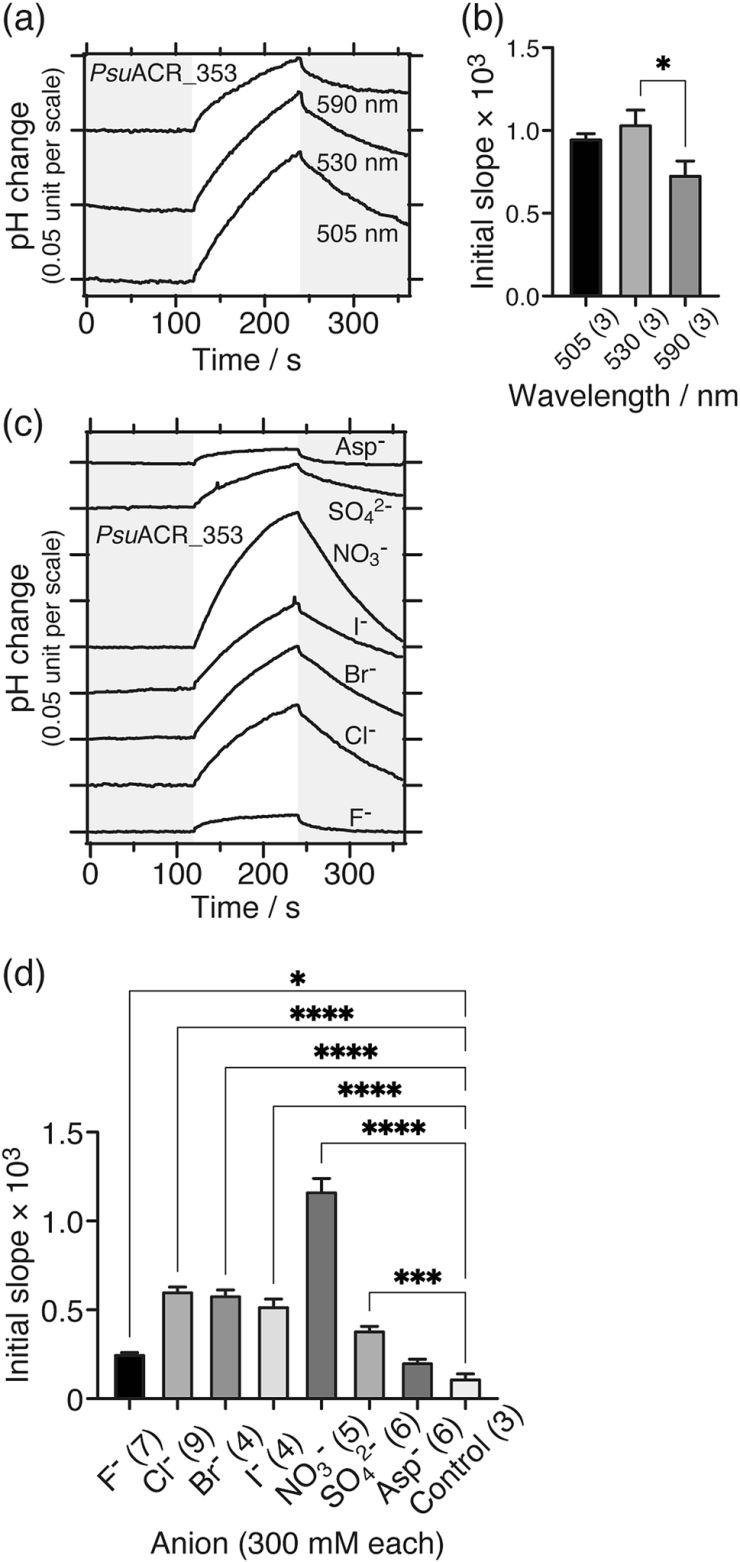
Anion transport activity of *Psu*ACR_353 measured using the pH electrode method. (**a**) Comparisons of Cl^**−**^ transport activities at various excitation light wavelengths (10 mW/cm^2^). LED lights were illuminated for 2 min as shown on a white background. (**b**) Statistical comparisons of Cl^**−**^ transport activities at various excitation light wavelengths. Data are reported as means and S.E.M.; the numbers in parenthesis indicate the numbers of independent experiments. One-way ANOVA followed by Tukey’s test was performed (*p* value; * 0.0471). (**c**) Comparisons of anion-dependent transport activities. 530 nm LED light (10 mW/cm^2^) was illuminated for 2 min as shown on a white background. (**d**) Statistical comparisons of anion-dependent transport activities. Data are reported as means and S.E.M.; the numbers in parentheses indicate the number of independent experiments. One-way ANOVA followed by Dunnett’s test was performed (*p* values; **** < 0.0001, *** 0.0002, * 0.0461). The negative control was yeast cells without integration of the *Psu*ACR_353 gene resuspended in 300 mM NaCl.

**Figure 5:**
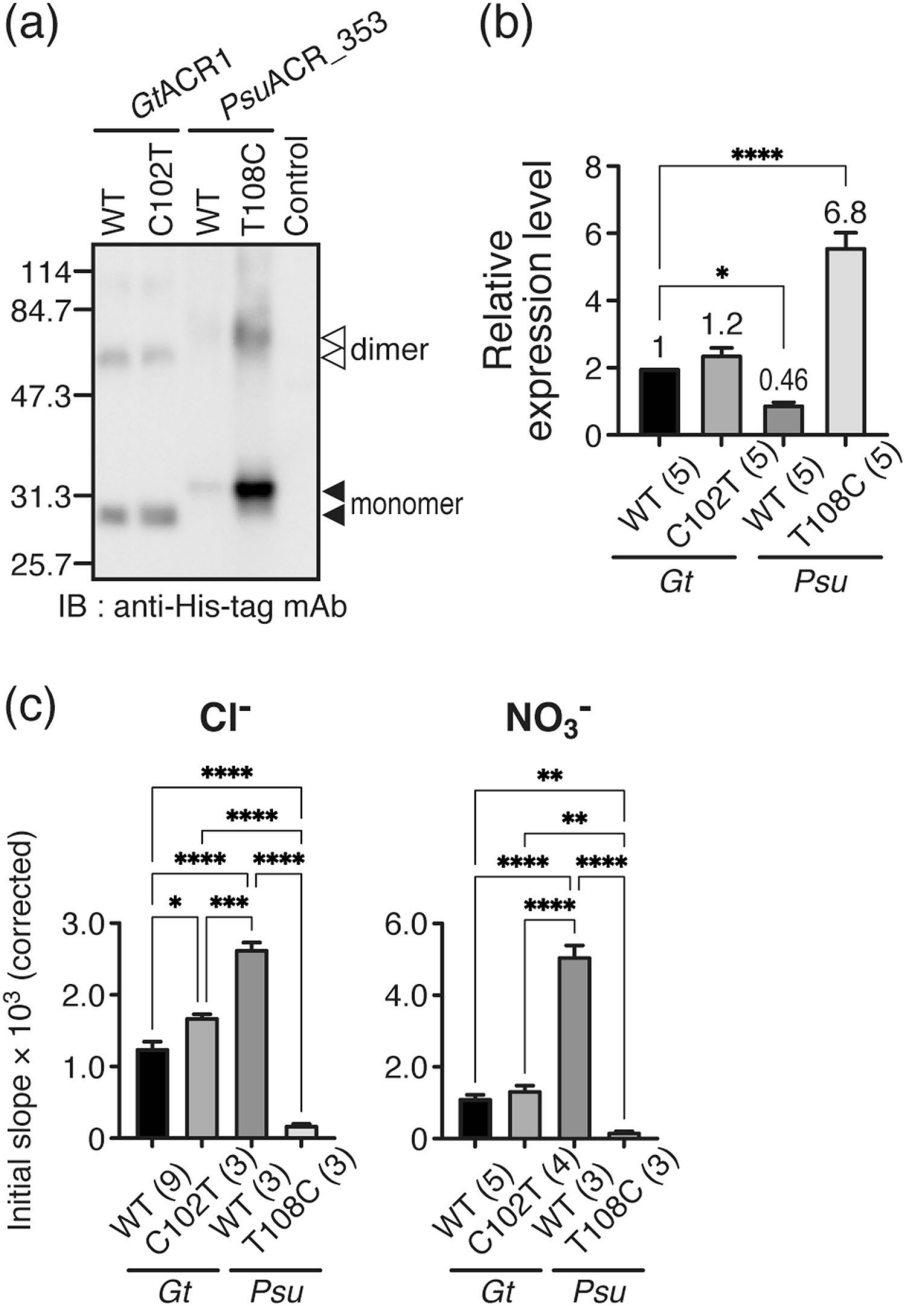
Quantitative comparisons of the anion transport activities of *Gt*ACR1, *Psu*ACR_353 and their mutants. (**a**) Western blotting using anti-His-tag mAb after SDS-PAGE; each ACR has monomer and dimer bands as indicated by filled and open triangles, respectively. The sum of band intensities was used to estimate protein expression levels. Original image of full-length membrane was shown in Supplementary Figure S5. (**b**) Statistical comparisons of relative expression levels. Data are reported as means and S.E.M.; the numbers in parentheses indicate the number of independent experiments. One-way ANOVA followed by Dunnett’s test was performed (*p* values; **** < 0.0001, * 0.0124). (**c**) Statistical comparisons of the transport activities of Cl^**−**^ (left) and NO_3_^**−**^ (right). Data were corrected by the relative expression level and are reported as means and S.E.M.; the numbers in parentheses indicate the number of independent experiments. One-way ANOVA followed by Tukey’s test was performed (*p* values; **** < 0.0001, *** < 0.0007, ** < 0.0049, * < 0.0492).

To characterize the anion transport activity of *Psu*ACR_353, we measured the action spectrum and anion dependence of its transport activity using the pH electrode method. Figure 4a shows that the Cl^**−**^ transport activities of *Psu*ACR_353 depend on the excitation light wavelength. Statistical comparisons of the initial slope amplitudes revealed that the activity at 530 nm was significantly larger than that at 590 nm (Fig. 4b). This result correlated well with a previous electrophysiological study^19^. We then measured the anion dependence of the transport activity of *Psu*ACR_353 using 530 nm light. Strong signals were observed not only for Cl^**−**^ but also for Br^**−**^, I^**−**^, NO_3_^**−**^ and even SO_4_^2**−**^ despite the low level of expression (Figs. 3b, 4c and 5b explained below). Especially for NO_3_^**−**^, the pH was increased by nearly 0.15 unit, which is ca. 3-times larger than even for the Cl^**−**^ transport of *Gt*ACR1 (Fig. 2f). In addition, statistical analysis clearly showed the strongest activity for NO_3_^**−**^, which was more than 2-times larger than that for the other anions (Fig. 4d). These results indicated that *Psu*ACR_353 had less anion selectivity as was *Gt*ACR1 (Fig. 2g), however *Psu*ACR_353 preferably transported NO_3_^**−**^.

To quantitatively compare the anion transport activities of *Gt*ACR1 and *Psu*ACR_353, their expression levels were estimated by Western blotting (Fig. 5a,b). The results showed that the relative level of expression of *Psu*ACR_353 was ca. 0.46-times smaller than that of *Gt*ACR1. Figure 5c and Supplementary Fig. S2 show statistical comparisons of the anion transport activities after correction to account for the expression levels of *Gt*ACR1 and *Psu*ACR_353. These results clearly showed that the transport activities of *Psu*ACR_353 for Cl^**−**^ and NO_3_^**−**^ were ca. 2- and 5-times larger than that for *Gt*ACR1, respectively, and therefore the preference for NO_3_^**−**^ of *Psu*ACR_353 was far greater than *Gt*ACR1 and other anion species.

### Anion transport activities of *Gt*ACR1-C102T and *Psu*ACR_353-T108C mutants

**Figure 6:**
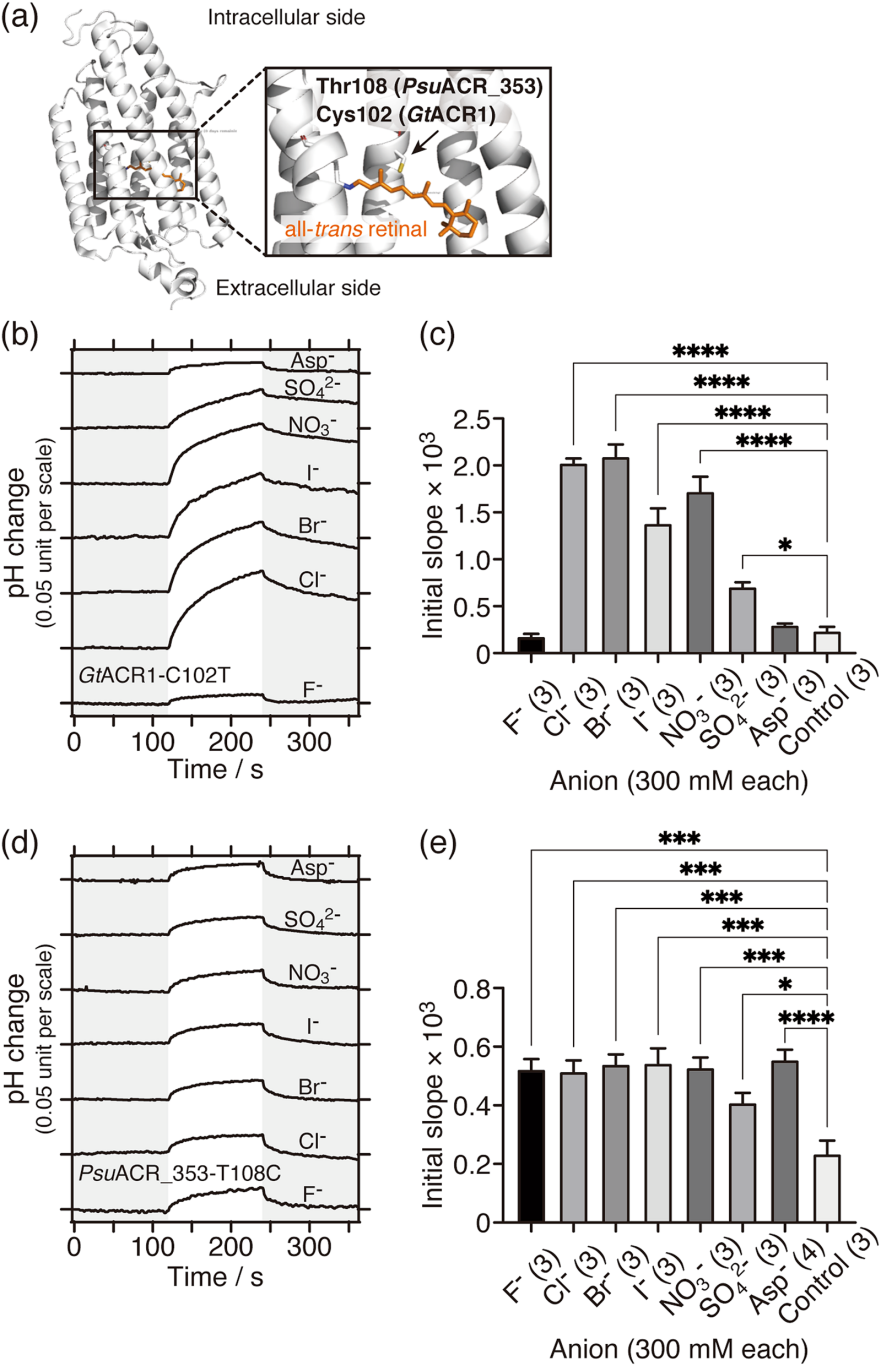
Anion transport activities of *Gt*ACR1-C102T and the *Psu*ACR_353-T108C mutants. (**a**) Location of the Cys102 residue in the *Gt*ACR1 structure (PDB ID 6CSM)^39^ drawn by PyMOL software. Cys102 and the all-*trans*-retinylidene chromophore are shown in stick representation by PyMOL. (**b, d**) Comparisons of anion-dependent transport activities of (**b**) *Gt*ACR1-C102T and (**d**) *Psu*ACR_353-T108C mutants. 530 nm LED light (10 mW/cm^2^) was illuminated for 2 min as shown on a white background. (**c, e**) Statistical comparisons of anion-dependent transport activities of (**c**) *Gt*ACR1-C102T and (**e**) *Psu*ACR_353-T108C mutants. Data are reported as means and S.E.M.; the numbers in parentheses indicate the number of independent experiments. One-way ANOVA followed by Dunnett’s test was performed (*p* values; for *Gt*ACR1-C102T **** < 0.0001, * 0.0197; for *Psu*ACR_353-T108C **** < 0.0001, *** < 0.0007, * 0.0338). Negative controls are yeast cells without integration of any ACR gene resuspended in 300 mM NaCl.

Finally, the anion transport activities of mutant ACRs were investigated using the pH electrode method. As described in the previous section, one difference in the amino acid sequence between *Gt*ACR1 and *Psu*ACR_353 is the amino acid conserved in the 3^rd^ transmembrane helix, i.e. *Gt*ACR1 has Cys102 whereas *Psu*ACR_353 has Thr108 at that position (Table 1, Figs. 3a, 6a, and Supplementary Fig. S1). We investigated the effects of that difference on anion transport activity, anion selectivity and preference of *Gt*ACR1-C102T and *Psu*ACR_353-T108C mutants using the pH electrode method.

For the *Gt*ACR1-C102T mutant (Fig. 6b,c), the intensities of the pH change and initial slope increased compared to wild-type *Gt*ACR1 (Fig. 2f,g). However, the anion dependence was almost the same as the wild-type (Fig. 6b,c). The expression level of the *Gt*ACR1-C102T mutant was also not significantly different from the wild-type (Fig. 5a,b). Taking this into account, a statistical comparison showed that the transport activities of anions other than F^**−**^ were almost the same as those of the wild-type (Fig. 5c and Supplementary Fig. S2). On the other hand, when compared with the transport activities of wild-type *Psu*ACR_353, the transport activities of the *Gt*ACR1-C102T mutant were significantly smaller and the preference for NO_3_^**−**^ was only slightly enhanced (Fig. 5c and Supplementary Fig. S2). Therefore, the Cys-to-Thr mutation at position 102 in *Gt*ACR1 resulted in almost no effect either on the anion transport activity or the selectivity or preference.

However, for the *Psu*ACR_353-T108C mutant (Fig. 6d,e), decreases in transport activities were observed for all anions by compared with wild-type *Psu*ACR_353 (Fig. 4c,d). In addition, the intensities of pH changes and initial slopes resulted in decreases to almost the same level (Fig. 6d,e), indicating that the anion dependence was completely lost due to that mutation. Taking into account the expression level of the *Psu*ACR_353-T108C mutant, which was about 7-times larger than wild-type *Gt*ACR1 (Fig. 5a,b), we concluded that the Thr-to-Cys mutation at position 108 in *Psu*ACR_353 caused a significant loss of function (Fig. 5c and Supplementary Fig. S2). These results indicate that the effects of the corresponding amino acids at positions 102 in *Gt*ACR1 and 108 in *Psu*ACR_353 on anion transport activity were completely different from each other, which would reflect the phylogenetic difference (Fig. 3a).

## Discussion

In this study, we used the pH electrode method to measure the anion transport activity of ACRs expressed as recombinant proteins in yeast cells. That method has already been established and used for ion pump-type microbial rhodopsins expressed in *E. coli* cells. Therefore, this is the first study that measured the activity of ion channel-type rhodopsins expressed in yeast cells. The advantages of this method include the simple instrumental setup and the use of a glass electrode pH meter, and the ease of measurement even for non-experts in electrophysiology. Indeed, electrophysiological techniques are powerful and are required for quantitative and precise measurements of ion channel functions. However, as we demonstrate here, the action spectrum and the substrate dependence of anion transport activity can be easily measured using the pH electrode method. We assume that this method will also be useful as a screening technique to explore and engineer ACRs with unique absorptions, anion selectivities, and so on.

We newly characterize the strong anion transport activity and NO_3_^**−**^ preference of *Psu*ACR_353 in this study. Govorunova et al. already performed electrophysiological measurements for various ACRs including *Psu*ACR_353^19^. However, no results were reported for such preferences probably because they did not perform the electrophysiological measurements using various anions other than Cl^**−**^. One reason for that we suppose is that the light-induced current of *Psu*ACR_353 caused by Cl^**−**^ transport was significantly smaller than not only the other ACRs tested but also for *Gt*ACR1^5,19^. That may be due to the much lower expression levels of *Psu*ACR_353 in their mammalian cell expression system because its expression level was also significantly small even in our yeast expression system (Fig. 5a,b). We noticed that the magnitude relationship of the Cl^**−**^ transport activities of *Psu*ACR_353 and *Gt*ACR1 reported by Govorunova et al.^5,19^ were completely different from our results obtained using the pH electrode method (Fig. 5c). This may also reflect differences in the expression levels of *Gt*ACR1 and *Psu*ACR_353 in each protein expression system.

On the other hand, the anion-dependent transport activity determined by each method can be considered equivalent. In the first report of *Gt*ACR1, its anion dependence was revealed by an electrophysiological method^5^. The result was that *Gt*ACR1 transported Cl^**−**^, Br^**−**^, I^**−**^ and NO_3_^**−**^ almost equally but transported F^**−**^ more weakly. In this study, we were successfully reproduced such a dependence using the pH electrode method (Fig. 2g). *Gt*ACR1 has less anion selectivity, which is similar to the anion pump rhodopsins but different from the potassium channel. It is speculated that a structure or a mechanism working as an anion selective filter is not necessarily sophisticated in *Gt*ACR1. Conversely, such a less anion selectivity may be connected to its biological roles in the algae *G. theta*, which awaits clarification in further studies. In contrast, *Psu*ACR_353 was revealed to transport NO_3_^**−**^ preferably to the other anions tested in this study (Figs. 4c, d, 5c and Supplementary Fig. S2). In the sense that *Psu*ACR_353 transported Cl^**−**^, Br^**−**^ and I^**−**^ almost equally, which was the same as *Gt*ACR1, and even SO_4_^2**−**^, *Psu*ACR_353 also showed less anion selectivity. However, the preference for NO_3_^**−**^ was far greater than the other anions. To our knowledge, such a preference has not been discovered in any microbial pump- or channel-type rhodopsins reported so far.

Nitrate (NO_3_^**−**^) is one of the most stable forms of nitrogen on earth and is used as a nitrogen source by most organisms including plants^32^. For NO_3_^**−**^ transport, both pump- and channel-type transporters exist in nature. Among them, a slowly activating anion channel 1 named SLAC1 and its homologous proteins named SLAH2 and SLAH3 expressed in plants are known to transport NO_3_^**−**^ preferably and take part in nitrate acquisition in roots, in nitrate translocation from roots to shoots, and in guard cell closure^32,34^. Therefore, *Psu*ACR_353 may play a role in NO_3_^**−**^ transport and might possibly take part in nitrate sensing and signaling, and furthermore, in the nitrogen assimilation in *P. sulcata* in nature. Indeed, it is known that *P. sulcata* senses the concentration of nitrate outside the cell and accumulates nitrogen as a form of protein-pigment, phycoerythrin, which contributes to the light-harvesting function for photosynthesis^35,36^. It should be noted that we tried to purify *Psu*ACR_353 to more deeply understand the mechanism and role of NO_3_^**−**^ transport using spectroscopic techniques, but those efforts failed because the protein was unstable in the presence of a detergent.

We wonder why *Psu*ACR_353 is able to preferably transport NO_3_^**−**^. One possible explanation is that the ionic radii or hydrated radii of anions contribute to the preference^37^. However, no sequential correlation is seen because the ionic and hydrated radii of NO_3_^**−**^ are 0.177 nm and 0.316 nm (Table 2), respectively, which is almost the same as those of Cl^**−**^ (0.180 nm and 0.319 nm, respectively). The crystallographic structure of *Gt*ACR1 in the dark state revealed that there is a continuous tunnel spanning through the protein, which is constructed by the 1st–3rd, and 7th transmembrane helices (Supplementary Fig. S1), and the radius of that tunnel is the same or smaller than the ionic radius of Cl^**−**^ (0.1–0.2 nm)^38,39^. Of course, a transient change of the tunnel radius accompanied by a structural change of the protein should also be considered because the structures of *Gt*ACR1 represent the state before light activation^38,39^, and the less anion selectivity of *Gt*ACR1 can be explained by the size of the tunnel radius and the ionic radius of anions. Another possible explanation is that when *Psu*ACR_353 transports NO_3_^**−**^ upon light activation, some spaces or sites are transiently formed, where NO_3_^**−**^ is preferably trapped or passes through, during the photocycle. Among the anions we tested in this study, only NO_3_^**−**^ is not spherically symmetrical. Thus, NO_3_^**−**^ has two hydrated radii, one for the axial radius and another for the equatorial radius (Table 2)^37^. Therefore, it is speculated that a possible mechanism for the NO_3_^**−**^ preference is that *Psu*ACR_353 transiently recognizes the asymmetrical structure of NO_3_^**−**^ and therefore transports NO_3_^**−**^ being hydrated.

**Table 2:** Summary of ionic and hydrated radii of anions tested in this study.

Anion	Ionic radius/nm	Hydrated radius/nm
F^**−**^	0.124	0.263
Cl^**−**^	0.180	0.319
Br^**−**^	0.198	0.337
I^**−**^	0.225	0.365
NO_3_^**−**^ mean	0.177	0.316
NO_3_^**−**^ axial	–	0.265
NO_3_^**−**^ equatorial	–	0.345
SO_4_^2**−**^	0.242	0.382

Data taken from^37^.

We suppose that Thr108 in *Psu*ACR_353 is one of the residues contributing to the NO_3_^**−**^ preference, which is based on comparisons of *Psu*ACR_353-T108C and *Gt*ACR1-C102T mutants (Figs. 5c, 6, and Supplementary Fig. S2). We investigated the effects of differences in amino acid residues at positions 102 and 108 in *Gt*ACR1 and in *Psu*ACR_353, respectively, on their anion transport activities, anion selectivity or preference for the first time in this study. Because the phylogenetic relationship between these ACRs is distant from each other (Fig. 3a), it is conceivable that the anion conducting paths and mechanisms in each ACR are different. The crystal structure of *Gt*ACR1 suggested that Cys102 is not involved in the anion conducting path^38,39^. On the other hand, in *Psu*ACR_353, Thr108 may be a part of a conducting path dedicated for NO_3_^**−**^.

## Conclusion

In this study, we successfully demonstrated the usefulness of the pH electrode method to measure the anion transport activity of ACRs. This method allows us to measure the action spectra and the anion dependence of the transport activity simply and easily, with results comparable to previous results obtained using electrophysiological methods. In addition, we identified the strong anion transport activity and the preference for NO_3_^**−**^ in *Psu*ACR_353 for the first time. Nitrate is one of the most stable forms of nitrogen and is used as a nitrogen source by most organisms including plants. Therefore, *Psu*ACR_353 may play a role in NO_3_^**−**^ transport and might take part in NO_3_^**−**^-related cellular functions in nature. Furthermore, we successfully demonstrated that Thr108, which corresponds to Cys102 in *Gt*ACR1, contributes to the NO_3_^**−**^ preference of *Psu*ACR_353. Such a preference has been discovered for the first time in microbial pump- or channel-type rhodopsins. This finding will be helpful to understand the anion transport mechanism of *Psu*ACR_353. We did not address the potential use of NO_3_^**−**^-preferred *Psu*ACR_353 for optogenetics because NO_3_^**−**^ is rare in mammalian cells. Possibly, *Psu*ACR_353 may be useful for optogenetics in plants.

## Methods

### DNA constructs of ACRs

Gene information about ACRs used in this study was obtained from GenBank and is summarized in Table 1. An 8 histidine tag was attached to the C-terminus of each ACR. The genes encoding ACRs with codon optimization for expression in *Pichia pastoris* were purchased from GENEWIZ (South Plainfield, NJ, U.S.A.). The procedures for constructing the pPICZ B vector (Thermo Fisher Scientific, Waltham, MA, U.S.A.) for *P. pastoris* were the same as our previous report^33^. The genes for *Gt*ACR1-C102T and *Psu*ACR_353-T108C mutants were prepared using PrimeSTAR Max DNA Polymerase (Takara Bio Inc., Shiga, Japan). The correctness of all nucleotide sequences was verified by dideoxy sequencing.

### Protein expression

The methylotrophic yeast *Pichia pastoris* SMD1168H strain (Thermo Fischer Scientific) was used as the protein expression host. The procedures for transformation of yeast and for the protein expression were almost the same as our previous report^33^. Briefly, transformed *P. pastoris* SMD1168H cells were pre-cultured in BMGY medium containing 100 μg/mL Zeocin (Thermo Fisher Scientific) for 1 day at 30 °C. The medium was exchanged to BMMY containing 100 μg/mL Zeocin, 2% (v/v) methanol (Fuji Film Wako Chemical Industries, Co. Ltd., Japan) and 20 μM all-*trans*-retinal (Sigma-Aldrich, St. Louis, MO, U.S.A.), and protein expression was induced for 1 day at 30 °C. The cells were collected by centrifugation (3,000 rpm, 5 min, 4 °C; himac CF16RN equipped with a T9A31 rotor; Hitachi Koki Co., Ltd., Tokyo, Japan).

### Anion transport activity measurement and data analysis

To measure anion transport activity, *P. pastoris* SMD1168H cells expressing ACRs were washed with 300 mM salt solution (NaCl, NaF, NaBr, NaI, NaNO_3_, Na_2_SO_4_, sodium aspartate; all from Fuji Film Wako Chemical Industries) 4 times by centrifugation (3,000 rpm, 5 min, 4 °C; himac CF16RN equipped with a T9A31 rotor). The cells were finally suspended in the same salt solution used for washing. As a negative control, *P. pastoris* SMD1168H cells without integration of any ACR gene were prepared. The optical density at 660 nm of each cell suspension was measured using a UV-1800 spectrophotometer (Shimadzu Corp., Kyoto, Japan) and adjusted to 1.9 on average. Anion transport activity was measured at temperatures below room temperature (16 °C on average) by monitoring pH changes using a LAQUA F-72 pH meter equipped with a standard ToupH pH electrode (HORIBA, Ltd., Kyoto, Japan). The initial pH was 5 on average. To measure the dependence of transport activity on the wavelength of light, blue-green (peak wavelength is 505 nm), green (peak wavelength is 530 nm) and orange (peak wavelength is 590 nm) LED light was illuminated. The light intensity was adjusted to 10 mW/cm^2^ on average, which was measured using a power meter (ORION, Ophir Optronics Solutions Ltd., Jerusalem, Israel). Anion transport activity was estimated by the initial slope of the first 10 s after LED light illumination for the time-dependent pH changes. More than three independent measurements were averaged. For statistical analysis, one-way ANOVA followed by Dunnett’s test and Tukey’s test were performed using GraphPad Prism 9 software (GraphPad Software, San Diego, CA, U.S.A.).

### SDS-PAGE and Western blotting

SDS-PAGE with 12% (v/v) polyacrylamide gels and Western blotting using an anti-His tag antibody conjugated with horseradish peroxidase (anti-His-tag mAb-HRP-DirecT, MBL Co., Ltd., Nagoya, Japan) were performed using standard protocols. *P. pastoris* cell samples were prepared as previously published^40^. Briefly, 1 mL of each cell suspension at an OD_660_ of ca. 1.9 was centrifuged to collect cell pellets. The wet weight of the cell pellets was 10 mg on average. The cell pellets were then resuspended in 1 mL 0.1 M NaOH and incubated for 5 min at room temperature. After centrifugation to remove the supernatant, the cell pellets were suspended in 250 μL SDS-PAGE sample buffer (60 mM Tris–HCl (pH 6.8), 2% (w/v) SDS, 5% (v/v) glycerol, 4% (v/v) β-mercaptoethanol and 0.0025% (w/v) bromophenol blue) and then boiled at 95 °C for 5 min. Finally, after centrifugation again, 6 μL of each supernatant diluted by one-sixth was loaded on the gel. To estimate the total amount of each ACR expressed in *P. pastoris* cells, the band intensities were analyzed using ImageJ software (U.S. National Institutes of Health, Bethesda, MD, U.S.A.) and 5 independent measurements were averaged. For statistical analysis, one-way ANOVA followed by Dunnett’s test was performed using GraphPad Prism 9 software (GraphPad Software).

## Supplementary Information

Supplementary Information
