# Supplementary material for: Preference of Proteomonas sulcata anion channelrhodopsin for NO3− revealed using a pH electrode method
Source: Sci Rep. 2021 Apr 12;11:7908. doi: 10.1038/s41598-021-86812-z (PMC8041784; doi:10.1038/s41598-021-86812-z)
Supplement: Supplementary file 1 — Supplementary Information [file 41598_2021_86812_MOESM1_ESM.docx]

**Supplementary Information**

**Preference of *Proteomonas sulcata* anion channelrhodopsin for nitrate revealed using a pH electrode method**

Chihiro Kikuchi, Hina Kurane, Takuma Watanabe, Makoto Demura,

Takashi Kikukawa & Takashi Tsukamoto

Contents:

**Supplementary Figure S1.** Amino acid sequence alignment of *Gt*ACR1

and *Psu*ACR_353

**Supplementary Figure S2.** Quantitative comparisons for the anion transport activities

of *Gt*ACR1, *Psu*ACR_353, and their mutants.

**Supplementary Figure S3.** Original image of the Western blot analysis.

**Supplementary Figure S4.** Absorption spectra of purified *Gt*ACR1 in the absence and

presence of CCCP.

**Reference**


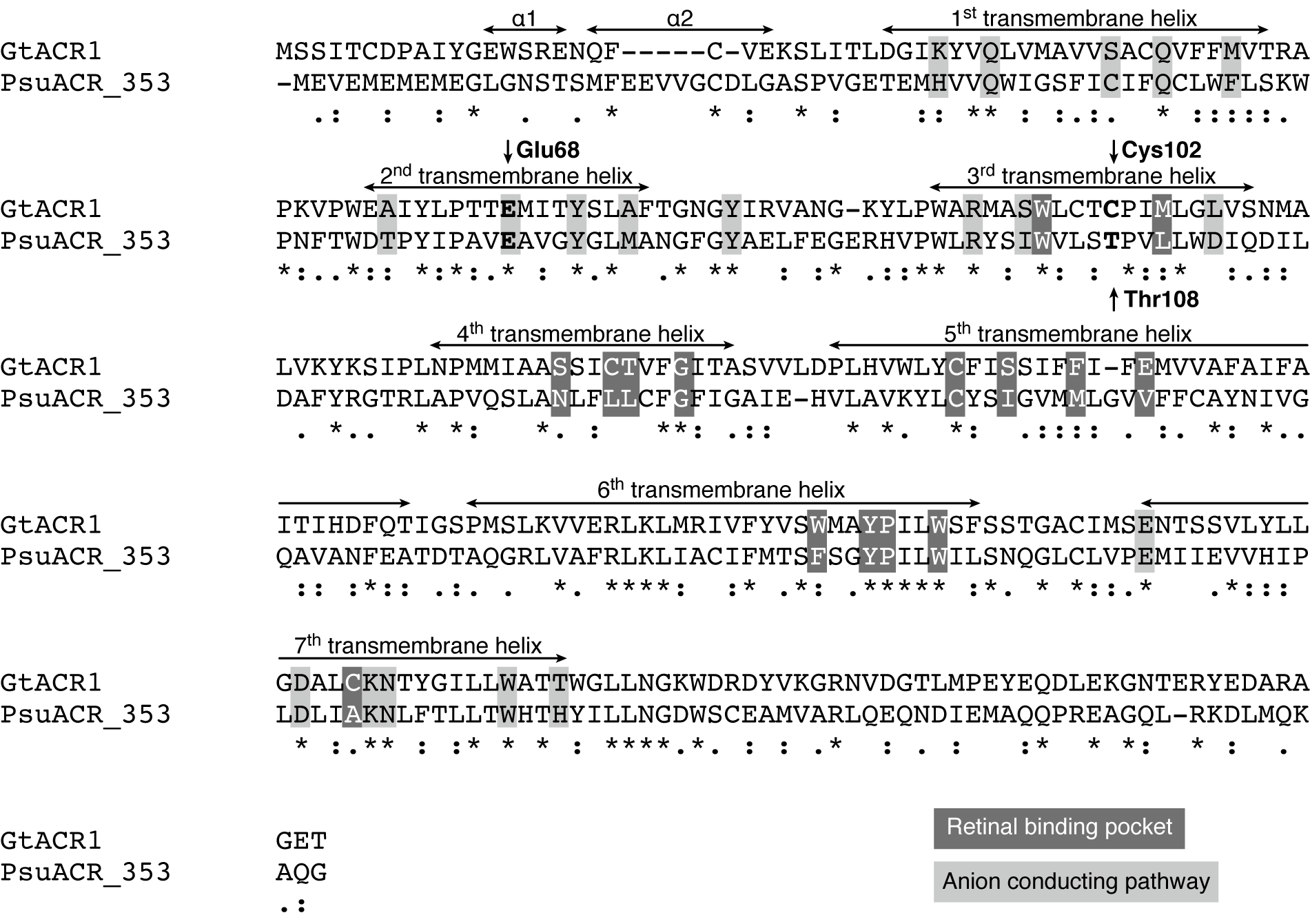


**Supplementary Figure S1.** Amino acid sequence alignment of *Gt*ACR1 and *Psu*ACR_353 made using the MUSCLE program (https://www.ebi.ac.uk/Tools/msa/muscle/). Amino acid residues composing the retinal binding pocket and the speculated anion conducting pathway are based on the crystal structure of *Gt*ACR1 (PDB ID: 6CSM) ^1^.


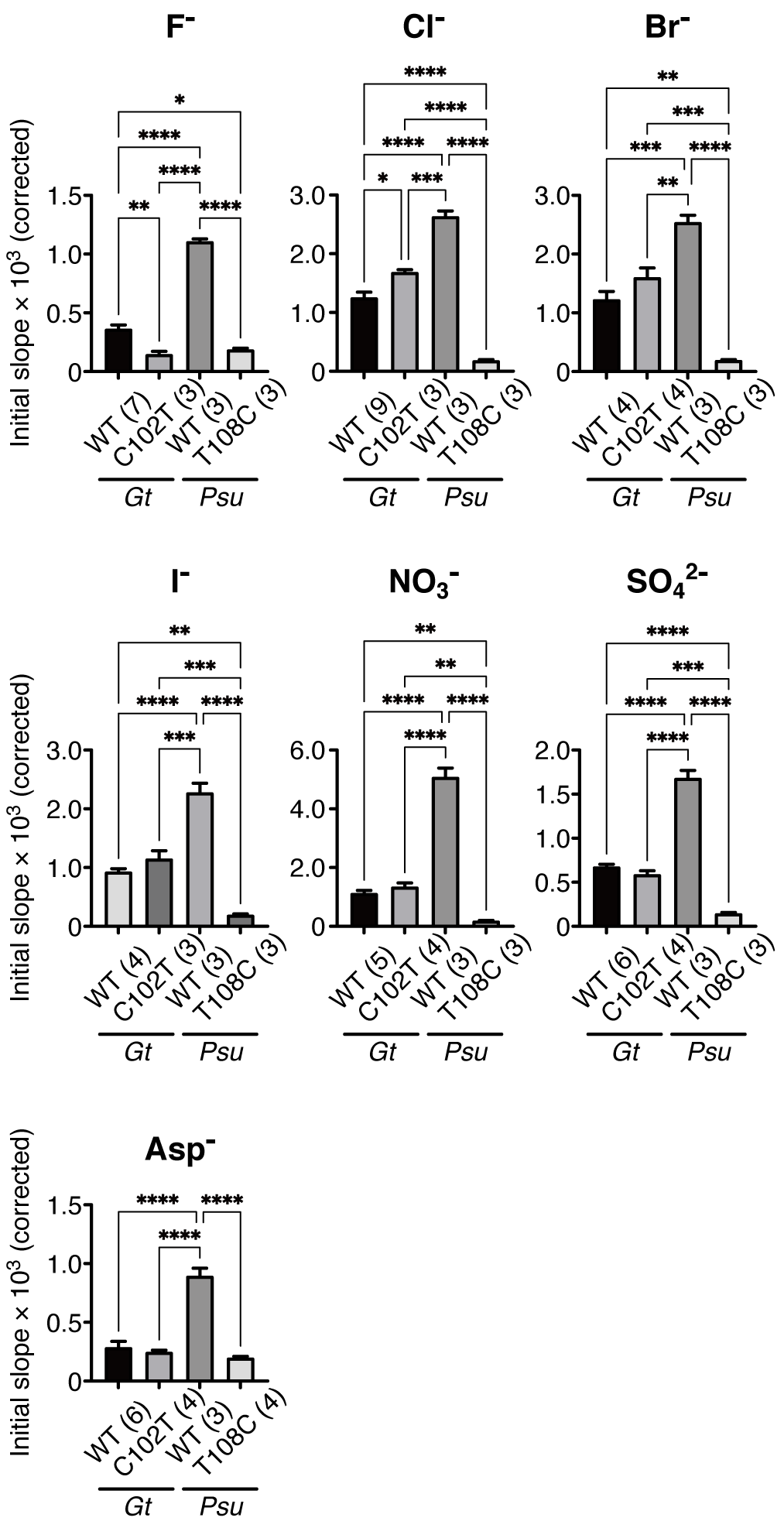


**Supplementary Figure S2.** Quantitative comparisons of the anion transport activities of *Gt*ACR1, *Psu*ACR_353, and their mutants. Data were corrected by the relative expression levels and are reported as means and S.E.M.; the numbers in parentheses indicate the number of independent experiments. One-way ANOVA followed by Tukey’s test was performed (p-values; **** < 0.0001, *** < 0.0007, ** < 0.0049, * < 0.0492).

**
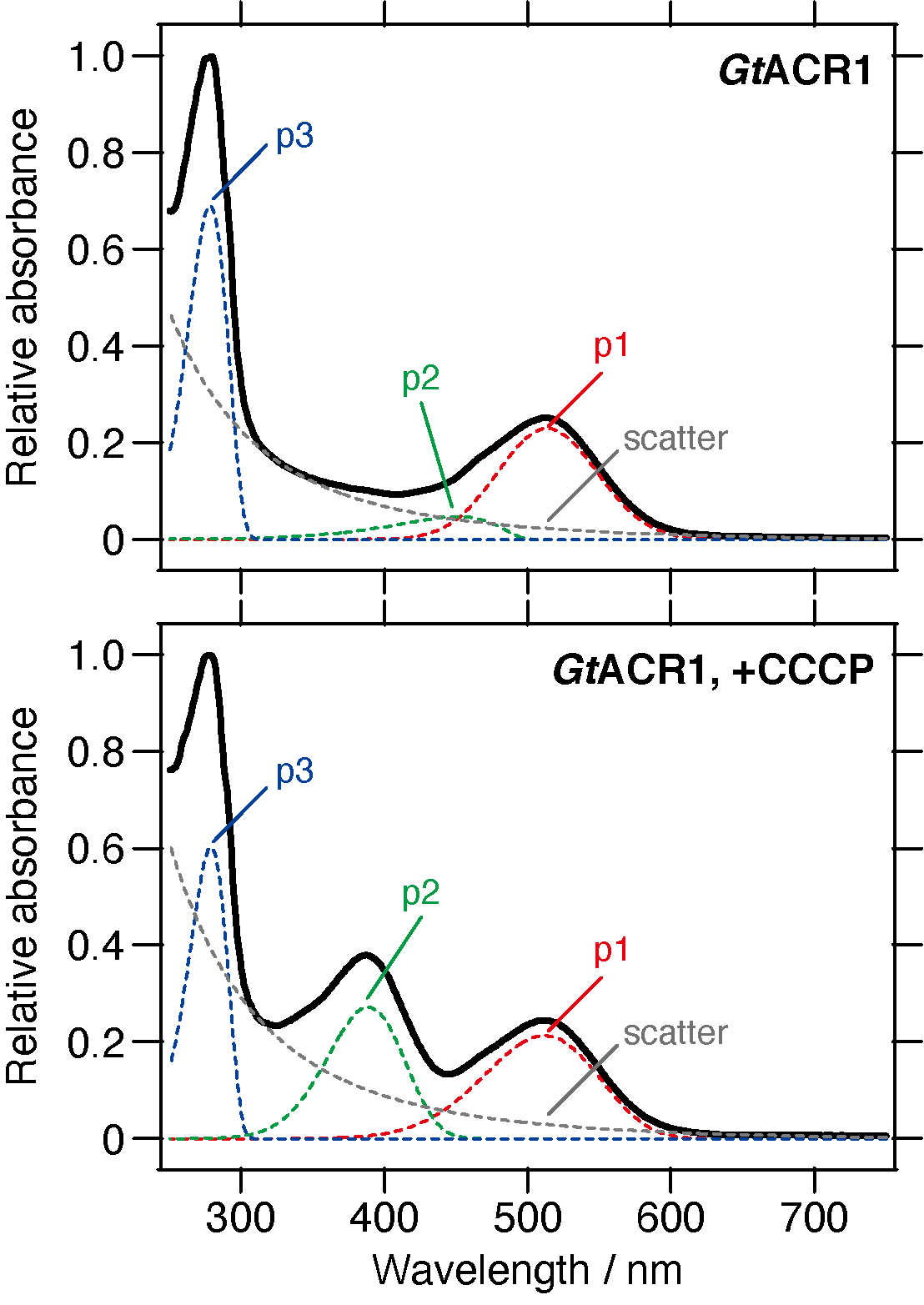
**

**Supplementary Figure S3.** Absorption spectra of purified *Gt*ACR1 in the absence (upper panel, black bold line) and presence (lower panel, black bold line) of CCCP. The spectra were decomposed into 4 terms using skewed Gaussian equation, spectral scattering (grey line), p1 (protonated retinal Schiff base, red line), p2 (deprotonated Schiff base and free retinal, green line), and p3 (aromatic amino acids, blue line), respectively. After the upper spectrum was measured, the lower spectrum was then measured by adding 10 μM CCCP. For the lower spectrum, the absorption spectrum of CCCP alone was subtracted as a baseline.

**
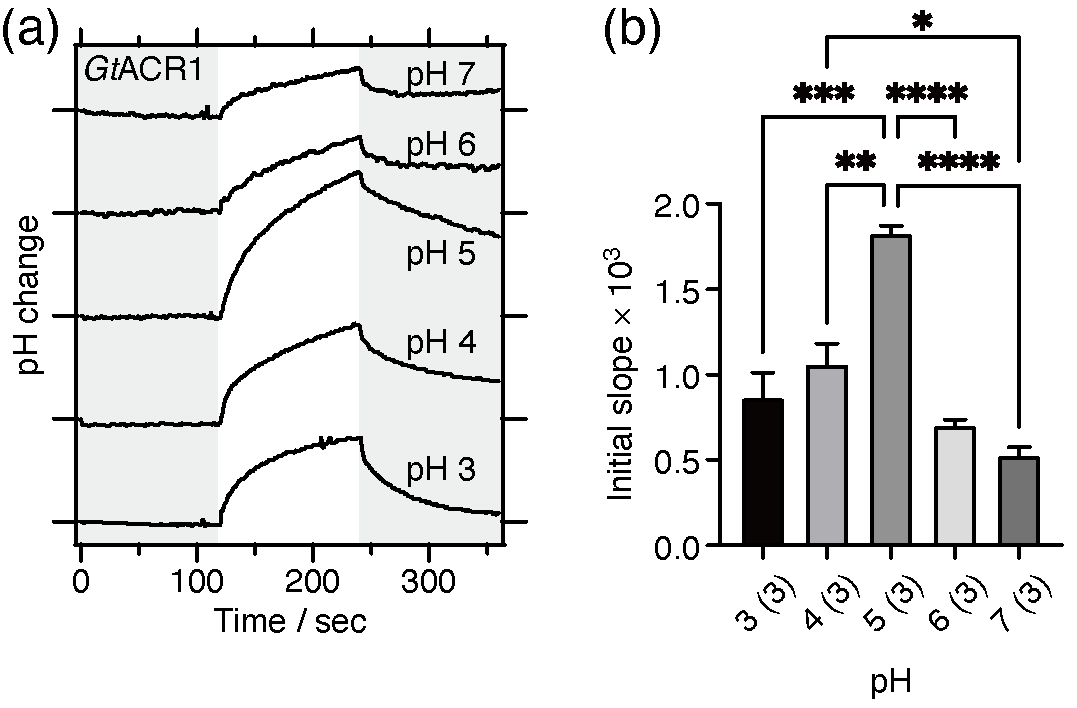
**

**Supplementary Figure S4.** The effect of pH on the Cl^-^ transport activity of *Gt*ACR1 measured using the pH electrode method. **(a)** Comparisons of the Cl^-^ transport activities of *Gt*ACR1 from pH 3 to pH 7. The pH was adjusted by the addition of very small amount of 0.05 M HCl and 0.05 M NaOH. 530 nm LED light (10 mW/cm^2^) was illuminated for 2 min as shown on a white background. **(b)** Statistical comparisons of the Cl^-^ transport activities at each pH condition. Data are reported as means and S.E.M.; the numbers in parentheses indicate the number of independent experiments. One-way ANOVA followed by Tukey’s test was performed (p-values; **** < 0.0001, *** 0.0002, ** 0.0011, * 0.0136).

**
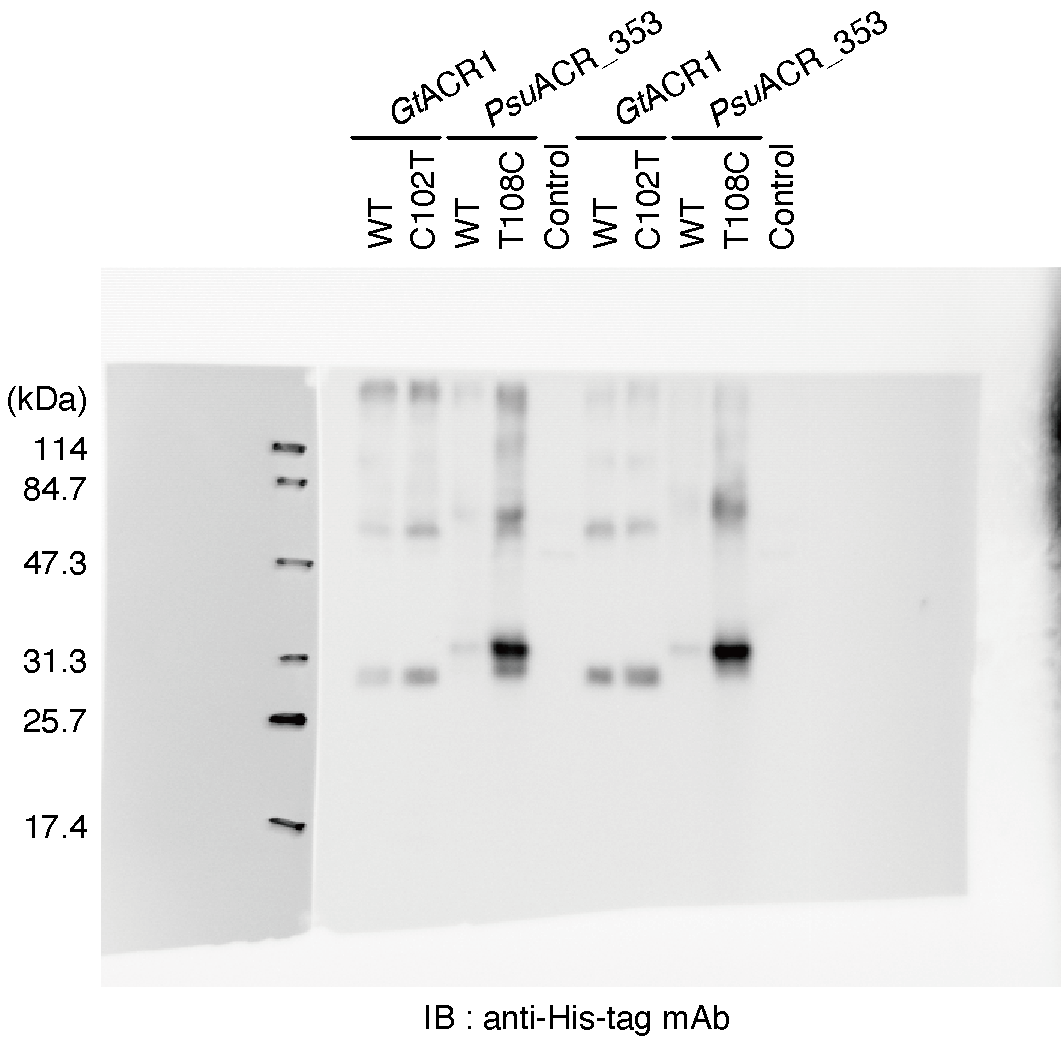
**

**Supplementary Figure S5.** Original image of Western blotting analysis shown in Figure 5a in the main text. Western blotting using anti-His-tag mAb after SDS-PAGE.

**Reference**

1. Kim, Y. S. *et al.* Crystal structure of the natural anion-conducting channelrhodopsin *Gt*ACR1. *Nature* **561,** 343–348 (2018).
